# Five pillars of centromeric chromatin in fungal pathogens

**DOI:** 10.1371/journal.ppat.1007150

**Published:** 2018-08-23

**Authors:** Vikas Yadav, Lakshmi Sreekumar, Krishnendu Guin, Kaustuv Sanyal

**Affiliations:** Molecular Mycology Laboratory, Molecular Biology and Genetics Unit, Jawaharlal Nehru Centre for Advanced Scientific Research, Jakkur, Bangalore, India; McGill University, CANADA

“*The greater the diversity, the greater the perfection*.”

—*Thomas Berry*

A centromere is classically defined as the primary constriction on a metaphase chromosome [1] that holds the sister chromatids together, binds to spindle microtubules, and brings about their separation during anaphase. Despite having a conserved and essential function, centromeres are among the fastest evolving DNA sequence loci in eukaryotic genomes [2]. With the advent of molecular biology techniques, centromeres could be mapped and sequenced in a large number of fungal species. The length of centromere DNA in fungi is found to be highly variable, classifying them as point (<400 bp), short regional (>400 bp, <20 kb), and large regional (>20 kb) [3]. Such diversity is achieved by different regulatory factors that have overlapping functions required for loading of the centromere-specific histone H3 variant centromere protein A/chromosome segregation 4(CENP-A/Cse4) to DNA to define centromere identity. Although genetic and epigenetic mechanisms of centromere formation across eukaryotes are largely conserved, there are examples of molecular innovation and genetic improvisation that help fungal species to maintain their ploidy across generations. In this review, we highlight five such genetic and epigenetic factors that define centromere identity in pathogenic fungi.

## DNA sequence and organization of DNA sequence elements

DNA sequence features provide the necessary template to act as a binding platform for kinetochore proteins. The genus *Candida*, which harbors several pathogenic species, presents a diverse array of centromere types. *Candida glabrata* carries point genetic centromeres, much like the 125-bp DNA sequence that serves as a fully functional point centromere in the budding yeast *Saccharomyces cerevisiae* [3–5]. Typically, genetic centromeres have specific and conserved DNA sequence motifs and confer mitotic stability to otherwise unstable plasmids carrying an autonomous replicating sequence (ARS) during cell division. Despite high-structural homology in DNA sequence elements, the point centromeres of *C*. *glabrata* are not fully functional in *S*. *cerevisiae*, suggesting that centromere function is species-specific [5, 6]. Short regional genetic centromeres of *Candida tropicalis* comprise a central core flanked by inverted repeats, similar to those of the fission yeast *Schizosaccharomyces pombe* [3, 7]. The sequence and orientation of these repeats are important for centromere function. Due to the presence of inverted repeats, the centromeres in *C*. *tropicalis* can acquire a hairpin loop-like secondary structure that might be crucial for kinetochore assembly. *Candida albicans* and *Candida dubliniensis*, on the other hand, possess unique and different centromere DNA sequences on each of their chromosomes [8, 9]. While *C*. *tropicalis* centromeres can stabilize an ARS plasmid, indicating a role of DNA sequence in centromere identity, the same does not hold true for *C*. *albicans*. *Cryptococcus neoformans*, a basidiomycetous pathogen that diverged from *C*. *albicans* more than 900 million years ago, harbors large regional centromeres that are rich in centromere-specific retroelements [10–12]. While the presence of such retroelements hints toward the functional dependence on DNA sequence in centromere function, more studies are needed to explore such links. Some fungal centromeres possess specific DNA sequence features. For example, *Candida lusitaniae* (teleomorph *Clavispora lusitaniae*) and *Malassezia sympodialis* centromeres are present in highly AT-rich regions of the genome but lack any easily detectable conserved sequence motifs or repeats [13, 14]. Whether any AT-rich DNA sequence can act as a centromere in these organisms, similar to what is observed in diatoms [15], remains an open question.

## Centrochromatin—CENP-A and chromatin modifications

The conserved centromere-specific histone H3 variant CENP-A/Cse4 is specifically present at all fungal centromeres identified to date but is largely excluded from other regions of the genome. *Mucor circinelloides* and *Phycomyces blakesleeanus* are notable exceptions in this regard, as they have no obvious CENP-A homologs, even though their centromeres are not yet physically mapped [16]. CENP-A is considered as the epigenetic determinant of centromere identity, as these molecules can seed the formation of a functional centromere in most organisms. This is supported by the fact that ectopic CENP-A incorporation can result in neocentromere formation, which is activated when an endogenous centromere becomes nonfunctional [17]. Although it is not well understood how CENP-A acts as the epigenetic determinant of the centromere, structural properties like a longer alpha N-terminal (αN) helix and the L1 loop region and biophysical features of the CENP-A nucleosome array, such as higher condensation properties, might be crucial for this role [18]. The process of CENP-A incorporation has been studied in *C*. *albicans*. Like other species, the CENP-A chaperone Holliday junction recognition protein/suppressor of chromosome mis-segregation 3 (HJURP/Scm3) is found to be crucial for CENP-A loading in *C*. *albicans* (our unpublished results). Regional centromeres harbor canonical histone H3 along with CENP-A. Post-translational modifications of histone H3 are crucial in forming a functional kinetochore [19]. Biochemical studies revealed the presence of heterochromatin histone marks such as dimethylation of histone H3 lysine 9 (H3K9diMe) across the centromeres of *C*. *neoformans* [10]. Apart from histone marks, DNA methylation has also been observed at the centromeres in *C*. *neoformans*, but its functional significance is unclear yet [10].

## Transcription and RNAi

For a very long time, the centromere locus was considered heterochromatic and transcriptionally inert. While centromere regions are generally transcription poor, landmark studies in several yeast species revealed that small interfering RNAs (siRNAs) derived from pericentromeric regions are necessary for centromere function [20, 21]. These studies indicated that centromere transcription is permissible and has a functional significance. A pan–fungal analysis of RNA interference (RNAi) proteins revealed that a few species, including *C*. *glabrata* and *Ustilago maydis*, have lost all of the proteins required for functional RNAi during the course of evolution, whereas species including *C*. *albicans* and *C*. *tropicalis* harbor a cryptic RNAi machinery [22]. A recent study in the pathogenic *Cryptococcus* species complex correlated the loss of RNAi with the length of centromeres, thereby proposing that RNAi helps in maintaining long repetitive, transposon-rich centromeres [10]. Whether the RNAi machinery has a functional significance in the centromere biology of this species complex and other fungal pathogens remains unexplored. Apart from RNAi, centromere transcription can also play a functional role through long noncoding RNA (ncRNA). Indeed, pervasive levels of transcription have been documented in various fungal species [23, 24]. However, the transcripts generated from the centromeres are significantly low in number compared to the rest of the genome, as shown in the *Cryptococcus* and *Ustilago* species complex [10].

## Replication and repair

Unlike metazoans, most fungal species have early replicating centromeres [25–27]. This temporally distinct replication timing not only allows better tolerance toward replication stress but also ensures proper kinetochore assembly at the centromeres. In *C*. *albicans*, centromeres replicate early in every synthesis phase (S-phase) and are associated with an early firing replicating origin [26]. Additionally, the formation of a neocentromere advances the replication time of the flanking region by activating an early replicating origin. This proximity effect was explained by a replication-coupled repair mechanism in a kinetochore-dependent manner [28]. Centromere-proximal origins stall randomly at the centromere, leading to accumulation of single-stranded DNA, which then recruits the homologous recombination proteins such as Rad51 and Rad52. These proteins physically interact with CENP-A in *C*. *albicans* and load it at the site of stalled replication forks; that is, centromeres. How this process is regulated to occur only during S-phase remains unknown, with possible implications for the CENP-A chaperone Scm3. Based on studies in many other nonpathogenic fungal species, the physical proximity of a partitioning locus (centromere) and an initiator site (replication origin) is relevant when one dissects the functional aspects of genome maintenance. However, evidence toward this connection is just beginning to emerge.

## Spatial location

Most fungal centromeres are clustered near spindle pole bodies (SPBs). This association may result in folding back of chromosomes and positioning them such that telomeres are juxtaposed in the interphase nucleus, giving rise to the Rabl conformation [29]. This phenomenon has been shown to occur in both animal and plant pathogens including *C*. *albicans*, *C*. *tropicalis*, and *Fusarium graminearum*. Using chromosome conformation capture (3C) experiments, clustered centromere DNA regions were shown to be present in close spatial proximity, leading to physical interactions between different centromeres [30–32]. It has been proposed that the clustering of centromeres aids in determining the site of centromere formation in these organisms. According to this hypothesis, a part of the nucleus is enriched with a pool of CENP-A proteins to form a CENP-A–rich zone or CENP-A cloud [33, 34]. It was proposed that the region of a chromosome that is near this CENP-A cloud would attract a higher level of CENP-A and thus serves as a preferred site for centromere formation. In *S*. *cerevisiae*, for example, a locally enriched population of accessory CENP-A molecules at pericentric chromatin has been shown to serve as a reservoir for rapid incorporation of CENP-A in the event of premature eviction from centromeres [35]. Further evidence supporting the CENP-A cloud hypothesis stems from studies in *C*. *albicans* in which neocentromeres were formed close to the native centromere in most cases [34]. In addition, neocentromeres in *C*. *albicans* change the spatial location to be a part of the centromere cluster by 3C experiments [31]. Interestingly, centromeres were found to be unclustered in premitotic *C*. *neoformans* cells that eventually cluster at the onset of mitosis [36]. Whether this centromere clustering also arises as a result of physical interactions among centromeres is not yet known.

**Fig 1:**
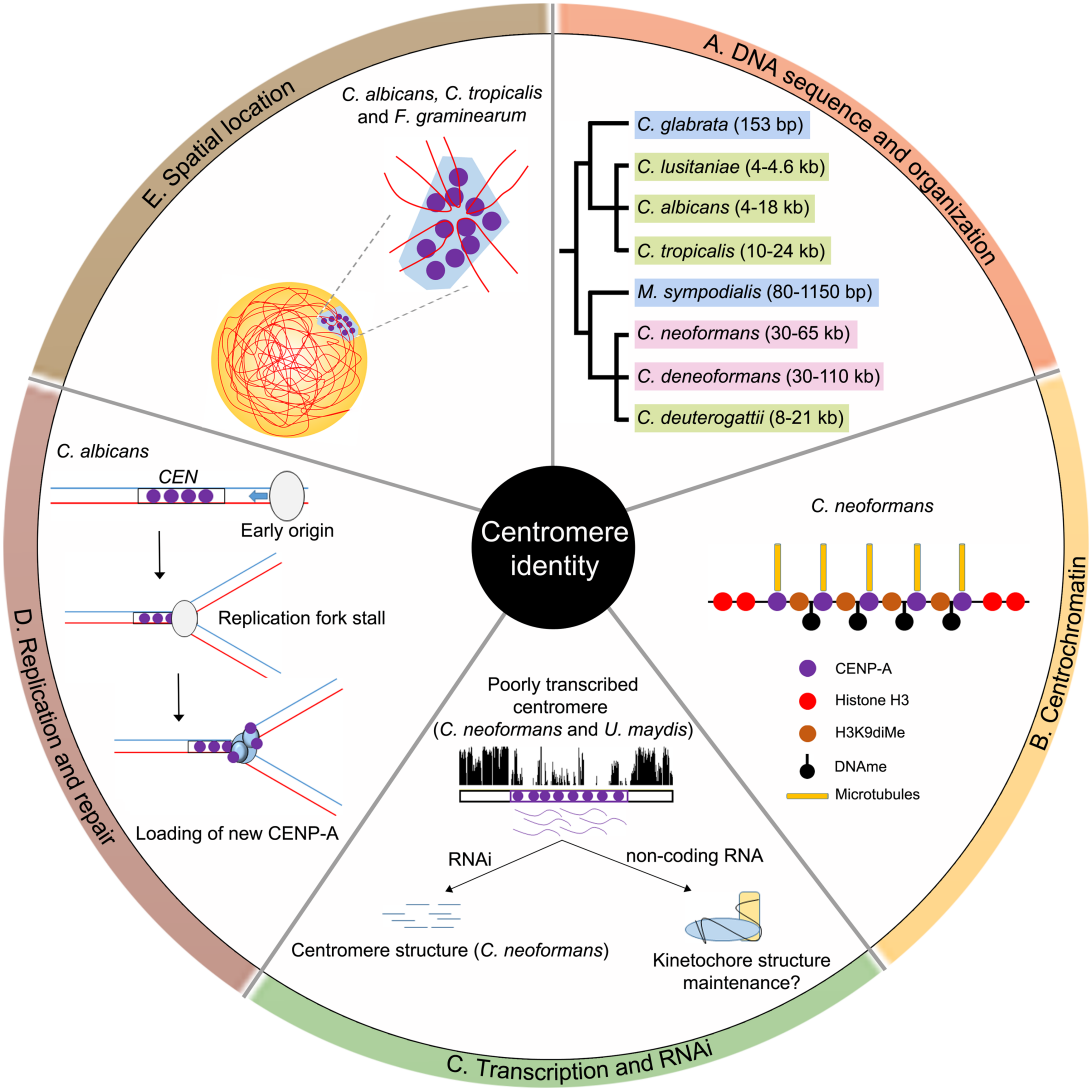
Five key determinants of centromere identity in pathogenic fungi. (A) The length and type of centromeres in various pathogenic fungi have been depicted in a cladogram. *Candida glabrata* and *Malassezia sympodialis* have point centromeres (blue); *C*. *albicans*, *C*. *tropicalis*, and *Cryptococcus deuterogattii* have small regional centromeres (green); and *C*. *neoformans* and *C*. *deneoformans* harbor large regional repetitive centromeres (pink). (B) Centromeric heterochromatin or centrochromatin refers to the various chemical modifications associated with the histones (both canonical and variant) as well as DNA at the centromeric locus. For example, centromere DNA is methylated at cytosines, and CENP-A nucleosomes are interspersed with H3K9-dimethylated nucleosomes in *C*. *neoformans*. (C) Transcription and a functional RNAi machinery at the centromere are required to preserve centromere identity in some organisms. The centromeres in the *Ustilago* and *Cryptococcus* species complexes are poorly transcribed. In *C*. *neoformans*, centromeric transcription is regulated by the RNAi machinery that silences centromeric retrotransposons to stabilize centromere structure. Unprocessed long noncoding RNA, whose function is unknown, is also produced from centromeres in this species. (D) DNA replication and repair proteins are known to play a key role in *C*. *albicans* centromere stability. The centromere proximal origins help in maintaining centromere function in this organism. Replication forks converging toward the centromere stall randomly, which leads to accumulation of single-stranded DNA (ssDNA) that recruits repair proteins like Rad51 and Rad52, along with new CENP-A molecules in *C*. *albicans*. (E) The spatial location of centromeres within the nucleus determines its activity and interaction with other nuclear subcompartments. All centromeres are clustered toward the nuclear periphery to form a CENP-A–rich zone in *C*. *albicans* and many other pathogenic fungi. This preferential spatial distribution helps to determine the site of centromere assembly in every cell cycle. CENP-A, centromere protein A; H3K9, histone H3 lysine 9; Rad, radiation sensitive; RNAi, RNA interference; ssDNA, single-stranded DNA.

Overall, here we summarize five key determinants among many that are needed for centromere identity in fungal pathogens (Fig 1). These factors may work sequentially or in parallel to ensure that centromere identity is maintained at all times in every cell cycle. For example, the repeats present at the centromere can form secondary structures, which may lead to double-stranded DNA breaks during replication and attract more CENP-A to these regions through DNA-repair pathways. The nuclear subdomain near SPBs is usually heterochromatic in nature, and the presence of the centromere cluster at a nuclear peripheral region contributes to poor transcription. Thus, a combination of factors can lead to more efficient CENP-A incorporation at the centromere. The CENP-A incorporation affects assembly of kinetochore proteins; for example, in *C*. *neoformans*, CENP-A assembly initiates the loading of other proteins, giving rise to a sequential kinetochore assembly in this organism [36]. On the contrary, *C*. *albicans* shows an interdependent kinetochore formation in which probably all kinetochore proteins assemble as a single complex [37]. However, our knowledge of the underlying mechanisms of CENP-A loading is still at its infancy, given the number of fungal species known to exist. The lack of knowledge is primarily due to difficulties associated with genetic manipulation of most pathogenic fungal species. Probing into the molecular mechanisms of centromere identity using diverse fungal species will yield significant insight into the structure-function-evolution of centromeres during speciation. Studies in *Candida* and *Cryptococcus* species complexes revealed that centromere-mediated recombination might have contributed to variations in the centromere structure and sequence [7, 10, 38]. Additionally, centromere sequences can also help in identification and classification of two or more closely related species. For example, *C*. *albicans* and *C*. *dubliniensis* centromere sequences show a high level of divergence while the rest of the genome sequences are highly homologous [9]. Discovery of highly efficient gene-targeting technologies such as the Clustered Regularly Interspaced Short Palindromic Repeats (CRISPR-Cas9) system for various *Candida* and *Cryptococcus* species as well as the application of efficient and improved DNA sequencing technologies for obtaining better genome assemblies are some of the significant recent developments that will advance pathogenic fungal molecular genetic research.
